# Monthly Patient Volumes of Buprenorphine-Waivered Clinicians in the US

**DOI:** 10.1001/jamanetworkopen.2020.14045

**Published:** 2020-08-24

**Authors:** Alexandra Duncan, Jared Anderman, Travis Deseran, Ian Reynolds, Bradley D. Stein

**Affiliations:** 1 Substance Use Prevention and Treatment Initiative, The Pew Charitable Trusts, Washington, DC; 2 Deerfield Institute, Deerfield Management Company, New York, New York; 3 Deerfield Management Company, New York, New York; 4 Now with Guidepoint, New York, New York; 5 RAND Corporation, Pittsburgh, Pennsylvania

## Abstract

This cross-sectional study examines the number of patients prescribed buprenorphine by US clinicians who were waivered to treat 30, 100, or 275 patients concurrently.

## Introduction

Buprenorphine, which is effective in treating opioid use disorder (OUD), can be prescribed for OUD by clinicians who obtain a US Drug Enforcement Administration (DEA) X-license after completing a Substance Abuse and Mental Health Services Administration (SAMHSA)–approved training. Federal regulations currently limit these waivered clinicians to treating 30, 100, or 275 patients with buprenorphine concurrently, with clinicians able to request an increased amount if they are limited to treating 30 or 100 patients. Clinicians historically treat fewer patients with buprenorphine than their limit allows.^1^ Given efforts to increase buprenorphine use, this cross-sectional study examines the number of patients treated by waivered clinicians.

## Methods

The IntegReview institutional review board determined that this cross-sectional study was exempt from review and that informed consent was waived because the study was a secondary analysis of existing data. Information on buprenorphine-waivered clinicians and patient limits was obtained from the DEA and SAMHSA in April 2019. Clinicians from the DEA file were matched by DEA numbers, name, location, and National Provider Identifier to calculate monthly clinician-level buprenorphine prescribing information from the Symphony Health Integrated Dataverse (eAppendix in the Supplement), a representative sample of US prescriptions. The data set used to perform this matching was derived by The Pew Charitable Trusts and Deerfield Management Company based on data provided by Symphony Health and other sources, covering the period of February 2017 to April 2019. Symphony Health data included DEA number, National Provider Identifier, and counts of patients, buprenorphine prescriptions, and prescription units. DEA- and Symphony Health–matched clinicians were then matched by name, DEA numbers, location, and phone and fax numbers to the April 2019 SAMHSA file and SAMHSA’s buprenorphine waiver verification website.^2^ To exclude clinicians unlikely to be responsible for the ongoing care of patients, such as those providing bridge prescriptions in an emergency department, we restricted our analysis to clinicians whose mean prescription supply was 7 or more days.

We calculated a monthly clinician-level patient census, covering the period from April 2017 to January 2019, of patients receiving buprenorphine for OUD, as well as the percentage of clinicians actively prescribing and the percentage of clinicians prescribing by patient limit. In the absence of the exact prescription fill date, we counted patients receiving a prescription supply of less than 30 days toward the clinician’s census in the month of the prescription, while we counted patients receiving a supply of 30 to 59 days in the month of the prescription and the subsequent month and patients receiving prescriptions for more than 59 days in the month of the prescription and 2 subsequent months. For patients carried over from a previous month, refilled prescriptions were subtracted to remove patients who would otherwise have been counted twice on a clinician’s census. Patients treated by multiple clinicians counted toward each clinician’s monthly mean. Analyses were performed in Excel 365 software (Microsoft) and SPSS statistical software version 20 (IBM) and were completed from June 2019 to January 2020.

## Results

Of 55 938 waivered clinicians identified, 28 448 (50.9%) wrote at least 1 buprenorphine prescription during the 22-month period (Table). The percentages for actively prescribing clinicians with 275 patients (4419 of 4507 [98.0%]) and actively prescribing clinicians with 100 patients (7504 of 8923 [84.1%]) were more than 2-fold the percentage for actively prescribing clinicians with 30 patients (16 525 of 42 508 [38.9%]). The median (interquartile range) monthly patient census was 101.5 (48.3-171.5) for the 275-patient clinicians, or 36.9.% of their patient limit; 23.9 (7.2-55.4) for the 100-patient clinicians, or 23.9% of their patient limit; and 3.4 (1.3-9.6) for the 30-patient clinicians, or 11.3% of their patient limit.

**Table.** Monthly Patient Census Among Active Clinicians Prescribing Buprenorphine for Opioid Use Disorder From April 2017 to January 2019

Patient limit	Waivered clinicians, No.	Active clinicians, No. (%)	Monthly patient census, median	Monthly patient census, percentile			
				10th	25th	75th	90th
30	42 508	16 525 (38.9)	3.4	1.0	1.3	9.6	21.6
100	8923	7504 (84.1)	23.9	2.0	7.2	55.4	99.6
275	4507	4419 (98.0)	101.5	16.1	48.3	171.5	257.3
Total clinicians	55 938	28 448 (50.9)	8.3	1.0	2.1	35.9	105.7

## Discussion

Consistent with earlier studies using nonnational data,^3^ this cross-sectional study found that approximately half of waivered clinicians prescribed buprenorphine and most treated at levels below their patient limits, although there remains tremendous unmet demand for OUD treatment.^4^ In 2016, active 100-patient clinicians were permitted to treat 275 patients after meeting specific criteria. Approximately half of clinicians with approval to treat 275 patients treated more than 100 patients concurrently, but most of those clinicians treated well below their patient limit.^3^ Our findings suggest the need for ongoing efforts to address treatment barriers, including policy efforts to minimize concerns about DEA oversight,^5^ increase clinician and care coordination reimbursement,^6^ and support waivered clinicians caring for patients.^3^

Limitations of this study include reliance on monthly prescriber-level prescriptions (eg, the month the prescription was dispensed, not a specific date), rather than patient-level claims, to calculate patient counts. Future work should examine factors associated with variations in patient census and clinician patient limits.

## Notes

Supplement. **eAppendix.** Data Sources
