# Supplementary material for: Monthly Patient Volumes of Buprenorphine-Waivered Clinicians in the US
Source: JAMA Netw Open. 2020 Aug 24;3(8):e2014045. doi: 10.1001/jamanetworkopen.2020.14045 (PMC7445589; doi:10.1001/jamanetworkopen.2020.14045)
Supplement: Supplement. — eAppendix. Data Sources [file jamanetwopen-3-e2014045-s001.pdf]

Supplementary Online Content

Duncan A, Anderman J, Deseran T, Reynolds I, Stein BD. Monthly patient volumes of buprenorphine-waivered clinicians in the US. *JAMA Netw Open*. 2020;3(8):e2014045. doi:10.1001/jamanetworkopen.2020.14045

eAppendix. Data Sources

This supplementary material has been provided by the authors to give readers additional information about their work.

© 2020 Duncan A et al. *JAMA Network Open.*

eAppendix. Data Sources

Information on clinicians' buprenorphine waiver status and their patient limit was derived from April 2019 files obtained from the Drug Enforcement Administration (DEA), the Substance Abuse and Mental Health Services Administration (SAMHSA) files, and the web-based SAMHSA Buprenorphine Lookup tool. We restricted our final population of waivered clinicians appearing in both a DEA and SAMHSA source as having the authority to prescribe buprenorphine for opioid use disorder (OUD).

The Symphony Health Solutions Integrated Dataverse (SHS), a representative sample of U.S. prescriptions, was the source of buprenorphine prescriptions from February 2017 – January 2019. The SHS dataset captures 92% of U.S. retail, mail order, and specialty pharmacy transactions and extrapolates these data to represent full U.S. coverage. National provider identifier (NPI), an identifier of clinicians reported in the National Plan and Provider Enumeration System (NPPES) database as well as supplementary identifiers like phone and fax numbers, were used to facilitate matching clinicians across datasets. The analysis was limited to buprenorphine for OUD products, excluding buprenorphine products indicated for pain. Buprenorphine for OUD indication was verified by a physician and in DailyMed, a list of drugs submitted to the Food and Drug Administration.

Matching clinicians across data sources

We used data from the DEA, SAMHSA, and SHS to identify buprenorphine waivered clinicians writing buprenorphine for OUD prescriptions. The DEA April 2019 file contained 58,107 unique DEA X-license numbers, indicating the clinician associated with the X-license is allowed to prescribe buprenorphine for OUD. The DEA file also included clinician name, additional DEA numbers, and geographic location. The SHS database contained a DEA and NPI number associated with all buprenorphine for OUD prescriptions dispensed from February 2017 - January 2019. However, as clinicians can have multiple DEA numbers,

© 2020 Duncan A et al. *JAMA Network Open.*

the DEA number in the SHS file may not be the waivered clinician's DEA X-license number. The SHS data also included clinician name, geographic location, clinician-level buprenorphine for OUD prescription count, prescription unit counts, and counts of patients receiving buprenorphine for OUD. The SAMHSA file of clinicians who had obtained a waiver authorized to write buprenorphine for OUD prescriptions (n=67,784) in April 2019 included clinician name and geographic location, was provided in response to a freedom of information act (FOIA) request, and was augmented with a search of the SAMHSA buprenorphine waiver lookup tool to ensure comprehensive coverage of the SAMHSA data.

Matching DEA and SHS

We first compared the clinicians who appeared in the April DEA file as having an X-license (n=58,107) with clinicians in the SHS file. To strengthen our matching efforts, we brought in related identifiers for each of the DEA X-licenses, the NPI number from the NPPES data and additional associated DEA numbers from the DEA file. Using the clinician's DEA X-license, NPI number, additional DEA numbers, as well as name and geographic location, we were able to match 30,046 clinicians appearing in both the DEA file and SHS file as having written at least buprenorphine prescription. There remained 28,061 clinicians with a DEA X-license from the DEA file who did not prescribe buprenorphine during the study period.

Matching DEA/SHS and SAMHSA

We next compared those with a DEA X-license who wrote at least one buprenorphine prescription (n=30,046), and those with a DEA X-license who did not prescribe (n=28,061) with SAMHSA data. We matched DEA/SHS to SAMHSA using DEA number, name, geographic location, phone number, and fax number. We were able to identify matches in the SAMHSA data for 98.8% (n=29,690) of the waivered

© 2020 Duncan A et al. *JAMA Network Open.*

clinicians who wrote at least one buprenorphine prescription and 97.2% (n=27,266) of the clinicians with a DEA X-license who did not prescribe during our time window, for a total of 56,956 clinicians.

<u>Calculating monthly buprenorphine for OUD patient census</u>

The SHS data contain clinician-level prescription count, prescription unit counts, and patient counts of buprenorphine for OUD. We used these variables to calculate a monthly clinician-level patient census of patients receiving buprenorphine for OUD. We sought to focus on prescribing in which the clinician was responsible for the patient's treatment episode. Therefore, we excluded clinicians whose average prescription length was less than 7 days, short term prescriptions likely to occur when a buprenorphine prescription is used for detox or to bridge a patient until they can engage in ongoing care, such as may often occur in emergency departments and in criminal justice re-entry situations. This resulted in the exclusion of 1,018 clinicians resulting in a final population of 55,938 clinicians.

Given that only the month but not the day the prescription was dispensed was available, patients receiving less than 30 days' supply counted towards the clinician census only in the month of the prescription; 30-59 days in the month of the prescription and subsequent month, and prescriptions for more than 59 days the month of the prescription and two subsequent months. This methodology required a two month lookback period, allowing us to assign patients in April 2017 who filled prescriptions in February 2017 and March 2017. Because of this lookback period, our study period became April 2017 – January 2019, resulting in 224 clinicians who wrote prescriptions in February 2017 - March 2017 but did not write prescriptions in April 2017 – January 2019 and are therefore non-prescribers in our analysis. For patients carried over from a previous month, refilled prescriptions were subtracted to remove patients that would otherwise have been counted twice on a clinician's census. Patients treated by multiple clinicians counted towards each clinicians' monthly census. The clinicians'

© 2020 Duncan A et al. *JAMA Network Open.*

average monthly patient census was calculated by dividing the number of patients on the panel each month from April 2017 through January 2019 by the number of months in which the clinician had patients.

We then calculated monthly patient census medians, the percentage of clinicians actively prescribing, and percentage prescribing by patient limit. Analyses were performed in Microsoft Excel for Microsoft 365 and IBM SPSS Version 20. IntegReview institutional review board determined that this cross-sectional study was exempt from review and that informed consent was waived because the study was a secondary analysis of existing data.

Limitations

The study findings must be considered within the context of its limitations. The study relied on monthly clinician-level prescription and patient counts, rather than patient-level claims. As a result, our assignment of patients to clinicians in each month is imprecise, but should not be biased. The clinician's patient limit was determined by their limit in April 2019, and clinicians may have had a lower patient limit during the observed study period. We used the number of prescription units dispensed to calculate the days supply, assuming the patient was supposed to take the same number of units every day. This approach could result in either an overestimate or underestimate of the days supply intended by the prescribing clinician. We assume that prescriptions of buprenorphine indicated for OUD were used to treat OUD, recognizing that in some cases such formulations are instead used to treat pain.

© 2020 Duncan A et al. *JAMA Network Open.*
